# Mechanical Fault Diagnostic in PMSM from Only One Current Measurement: A Tacholess Order Tracking Approach

**DOI:** 10.3390/s20175011

**Published:** 2020-09-03

**Authors:** Abdallah Allouche, Erik Etien, Laurent Rambault, Thierry Doget, Sebastien Cauet, Anas Sakout

**Affiliations:** 1 LaSIE Laboratory UMR CNRS 7356, University of La Rochelle, 17042 la Rochelle, France; asakout@univ-lr.fr; 2 LIAS Laboratory, University of Poitiers, 86073 Poitiers, France; erik.etien@univ-poitiers.fr (E.E.); laurent.rambault@univ-poitiers.fr (L.R.); thierry.doget@univ-poitiers.fr (T.D.); sebastien.cauet@univ-poitiers.fr (S.C.)

**Keywords:** PMSM diagnostic, variable speed, tacholess order tracking, motor current analysis, statistical approach

## Abstract

This article presents a mechanical fault diagnosis methodology in synchronous machines using only a single current measurement in variable speed conditions. The proposed methodology uses order tracking in order to sample the analysis signal as a function of the rotor angle. The spectrum of the signal is then independent of speed and it could be employed in frequency analysis. Order tracking is usually applied using rotor position measurement. In this work, the proposed method uses one current measurement to estimate the position as well as the analysis signal (rotation speed). Furthermore, a statistical approach is used to create a complete diagnosis protocol. At variable speed and with only one current measurement the diagnosis is challenging. However, order tracking will allow simpler analysis. The method is proved in simulations and experimental set-up.

## 1. Introduction

In this article, we present a methodology for diagnosing mechanical faults for synchronous machines used in non-stationary conditions (variable speed). The procedure is developed from a single current measurement and without a speed sensor. Many methods have been developed for the diagnosis of electrical machines from Electrical Signature Analysis (ESA) [1,2]. Their adaptation to the case of variable speed requires the use of specific signal processing [3,4,5] or methods based on sensorless control theory [6,7]. In this article, the method used is the Tacholess Order Tracking (TOT) [8,9]. It is based on the sampling of measurements with respect to an angle (mechanical angle or electrical angle). The advantage of this approach is to be able to stationarize the spectral representation, that is to say, to make the spectrum of the signal independent of the speed of rotation and thus to facilitate the analysis.

It is now well established that instantaneous speed is a good candidate for the diagnosis of mechanical faults in electrical machines [10]. In the case of synchronous machines, this speed is proportional to the frequency of the electrical signals. Consequently, Motor Current Signature Analysis (MCSA) methods have been highly developed [11,12,13]. For its part, TOT is generally implemented using two complementary measures: The first contains the signature of the defect sought, and the second is used to estimate the mechanical angle [14,15,16]. The originality of this work is to propose a software sensor allowing, from a single measurement, to extract the quantity possessing the fault signatures, to estimate the mechanical angle of the machine, and to carry out the angular sampling. It is a preparatory work for the design of a new software sensor that can be used in industry and the main constraint is that all of these operations be carried out in real-time.

This article proposes a complete procedure (measurement, processing, angular sampling, and fault detection procedure). It follows on from the work in [17] by analyzing more precisely the design of the PLL, introducing a new online angular sampling tool, and showing that the TOT allows a very simplified statistical analysis. The procedure is tested in simulation and on a laboratory test bench.

The article is organized into six sections. Section 2 recalls the modulation phenomenon occurring in currents in the presence of faults. Section 3 presents the angular resampling procedure based on an original PLL design. The experimental results obtained on the test bench are presented in Section 4 and discussed. In Section 5, the resampled signal is exploited by a statistical diagnostic procedure. It is shown that this procedure is simplified compared to a classical temporal sampling.

## 2. Electrical Currents Analysis

Electrical currents analysis focuses on diagnosis of electrical machines (asynchronous and synchronous) from only currents measurements. In order to understand the fault signatures, a commonly accepted approach considers that mechanical faults will cause either a variation of torque or a variation of eccentricity. These variations cause phase modulations (PM) of current in case of torque variations and amplitude modulations (AM) in case of dynamic eccentricity variations [18,19]. The general form of the stator current can be expressed as

(1) I(t)=A(t)cos(θ(t))

where A(t) and θ(t) represent, respectively, the instantaneous amplitude and the instantaneous phase. In the case of a sinusoidal perturbations, the expressions of the modulated current are given as follows,

(2) $$A\left(t\right)=I\cdot [1+\alpha cos\left(2\pi f_{AM}t+\phi_{AM}\right)]$$

(3) $$\theta \left(t\right)=2\pi f_{s}t+\beta cos\left(2\pi f_{PM}t+\phi_{PM}\right)+\phi_{I}$$

where α, $f_{AM}$, and $\phi_{AM}$ represent, respectively, the modulation index, the frequency of the modulating signal, and the initial phase for AM modulation (as well as β, $f_{AM}$, and $\phi_{I}$ for FM modulation).

The isolation of fault components in currents is more difficult than in vibratory measurement because the signal-to-noise ratio is much lower and the fundamental electrical component is of high amplitude and masks the sought frequencies. One possibility is to remove this fundamental in order to amplify the fault frequencies [20]. However, the simplest solution is to demodulate the currents in amplitude or in phase. When the three currents are available, the concordia transform makes it possible to carry out these demodulations in a relatively simple manner. From a single current measurement, it is natural to use the Phase-Locked Loop (PLL) resulting from telecommunications [21]. In the field of electrical engineering, PLLs have been widely used in the analysis of electrical networks [22,23,24]. For synchronous motor, PLLs are exploited as virtual speed sensors from currents measurements [25,26]. In the following, a PLL is specifically designed for operations at variable frequency and amplitude to simultaneously estimate the rotation frequency and the mechanical position.

## 3. A PLL-Based Online Resampling

### 3.1. PLL Design and Improvements

**Figure 1:**
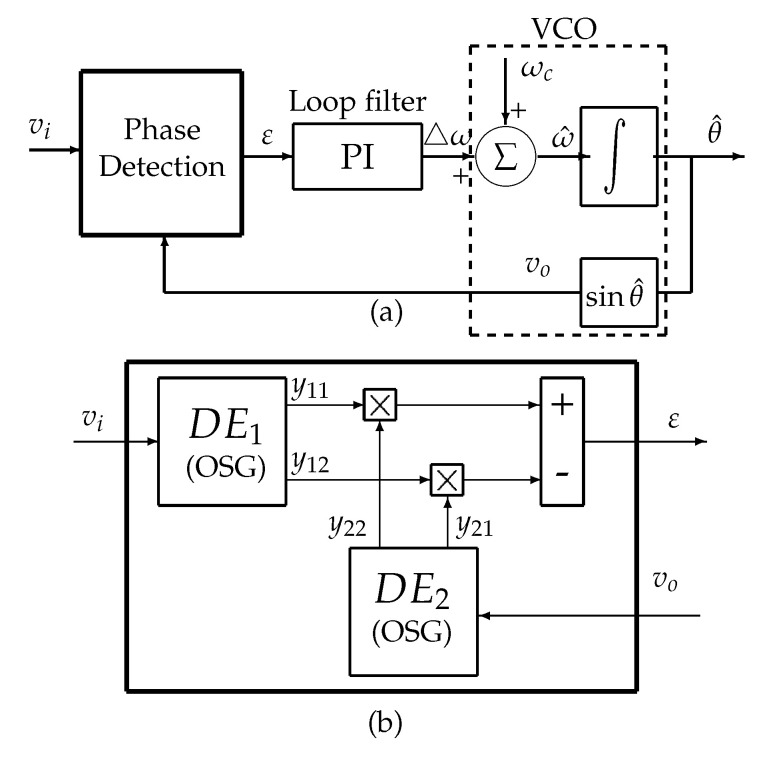
DE_PLL. (**a**) Basic structure. (**b**) Phase detector.

A classic PLL has three parts: phase detector (PD), loop filter (LF), and a voltage-controlled oscillator (VCO), Figure 1a. A simple multiplier could be used as a PD. However, higher harmonics will be generated [22]. To overcome this issue, an orthogonal signal generator (OSG) is used to generate two orthogonal signals. Due to orthogonality, harmonics in the output of PD are now eliminated.

In [22], an OSG-PLL based on derivative elements (DE) is proposed. Figure 1b shows the PD of the proposed PLL. Every DE is defined by two filters:

(4) $$DE\;\left\{\begin{array}{c} G\left(s\right)=\frac{\omega_{R}^{2}s}{s^{2}+2\omega_{R}s+\omega_{R}^{2}}\;\;bandpass\\ G^{{}^{\prime }}\left(s\right)=\frac{\omega_{R}^{2}}{s^{2}+2\omega_{R}s+\omega_{R}^{2}}\;\;low-pass \end{array}\right.$$

**Figure 2:**
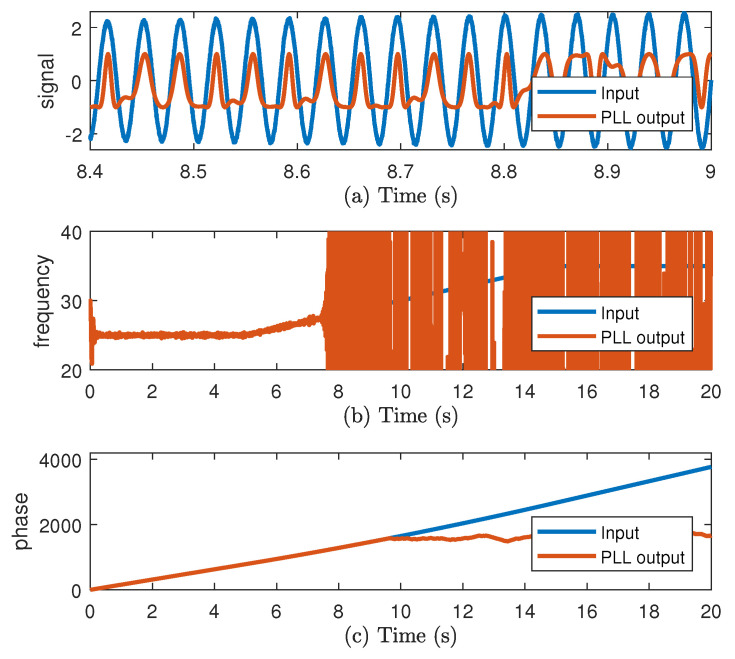
(**a**) Input and Phase-Locked Loop (PLL) sinusoidal output $v_{f}$, (**b**) frequency, and (**c**) phase.

The proposed structure is vulnerable to noise and to high variations of instantaneous amplitude of input signal. In order to show the behavior of this structure in those two conditions, the PLL is tested in simulation. The input is a sinusoidal signal where its amplitude and frequency change with time. In Figure 2, the input signal and the PLL estimations are shown. In Figure 2a, the instantaneous amplitude is increasing. In Figure 2b, at the start the frequency is estimated, but some noise is presented. Next, the estimation is lost because of the increasing amplitude as well as for the phase in Figure 2c.

**Figure 3:**
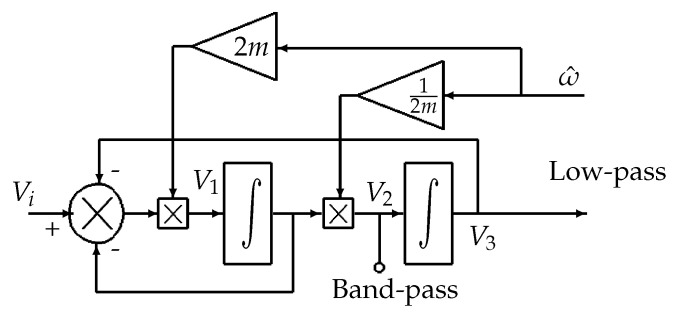
State variable structure.

The structure of the PLL is modified in order to have a better performance. The first improvement is to use adaptive filters. The state variable structure shown in Figure 3 could create two filters: bandpass and low-pass. The filters are tuned using the estimated instantaneous frequency.

When the damping ratio is set to m=1/2, the transfer functions of the new filters are as follows,

(5) $$\;\left\{\begin{array}{c} G_{x}\left(s\right)=\frac{\omega_{R}s}{s^{2}+\omega_{R}s+\omega_{R}^{2}}\;\;band-pass\\ G_{x}^{{}^{\prime }}\left(s\right)=\frac{\omega_{R}^{2}}{s^{2}+\omega_{R}s+\omega_{R}^{2}}\;\;low-pass \end{array}\right.$$

with $\omega_{R}$ representing at the same time the central frequency of the band-pass filter and the cut-off frequency of the low-pass filter. Here, $\omega_{R}$ is the estimated frequency $\hat{\omega }$.

**Figure 4:**
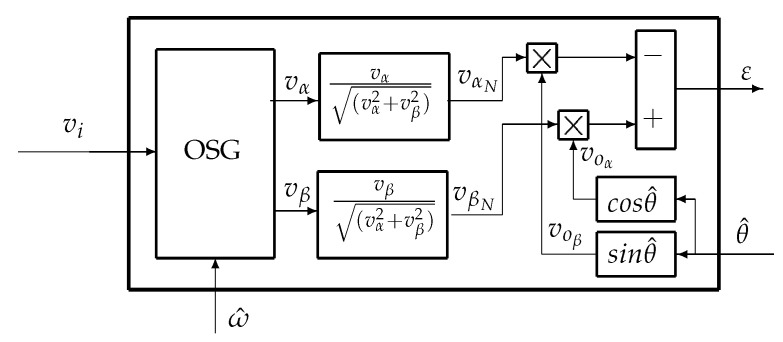
Normalization of input signal.

The second improvement is to normalize the amplitude of the input signal to ±1. The two orthogonal signals generated by the adaptive filters $v_{\alpha }$ and $v_{\beta }$ are used to create the normalized signals $v_{\alpha N}$ and $v_{\beta N}$, see Figure 4.

**Figure 5:**
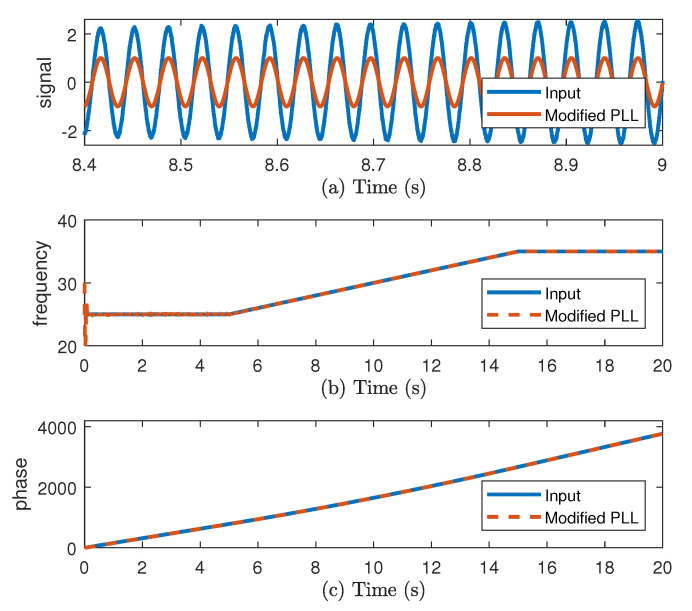
(**a**) Input and PLL sinusoidal output $v_{f}$, (**b**) frequency, and (**c**) phase.

After the two improvements, the PLL is tested using the same simulation as before. The estimated signal, frequency, and phase are shown in Figure 5. The frequency is estimated with reduced amount of noise. The estimation is not lost even with increasing amplitude, see Figure 5c.

### 3.2. Parameter Setting

In order to initialize the parameters of the PLL, the linearized model of the PLL is calculated. The error of the PD is as follows,

(6) $$\epsilon \approx k_{pd}\left(\hat{\theta }-\theta_{i}\right)$$

where $\hat{\theta }$ is the estimated phase and $\theta_{i}$ is the phase of the input signal.

The Proportional-Integral (PI) controller used has the following equation,

(7) $$f\left(s\right)=k_{p}+\frac{k_{i}}{s}$$

where $k_{p}$ and $k_{i}$ represent the proportional and the integral gains, respectively.

According to the work in [22], the closed-loop transfer function of the linearized model is as follows,

(8) $$H_{CL}\left(s\right)=\frac{\hat{\theta }\left(s\right)}{\theta_{i}\left(s\right)}=\frac{K_{p}K_{pd}s+K_{i}K_{pd}}{s^{2}+K_{p}K_{pd}s+K_{i}K_{pd}}$$

with $k_{pd}$ the PD gain is equal to $\omega_{s}/4$ or the PD is modified, and the new gain $k_{pd}$ should be calculated.

In Equation (5), if *s* is replaced with jω the transfer functions will be

(9) $$G_{x}\left(j\omega \right)=\frac{j\omega .\omega_{R}}{-\omega^{2}+j\omega .\omega_{R}+\omega_{R}^{2}};\;\;G_{x}^{{}^{\prime }}\left(j\omega \right)=\frac{\omega_{R}^{2}}{-\omega^{2}+j\omega .\omega_{R}+\omega_{R}^{2}}$$

If the input is sinusoidal at angular frequency $\omega =\omega_{0}$, and because the filters are adaptive through the estimated frequency, with a locked PLL $\omega_{R}≅\hat{\omega }≅\omega_{0}$ the filters will be

(10) $$G_{x}\left(j\omega_{0}\right)=\frac{j\omega_{0}^{2}}{-\omega_{0}^{2}+j\omega_{0}^{2}+\omega_{0}^{2}}=1;\;\;G_{x}^{{}^{\prime }}\left(j\omega_{0}\right)=\frac{\omega_{0}^{2}}{-\omega_{0}^{2}+j\omega_{0}^{2}+\omega_{0}^{2}}=-j$$

The last equation shows that those two filters have at $\omega =\omega_{0}$ a gain equal to 1 and phase shift equal to π/2.

After normalization of the input signal and because of using filter with gain = 1, we can exclude the two other OSG filters named $DE_{2}$ in Figure 1. The creation of two orthogonal signals of the estimated output is done simply by the functions Sin and Cos as shown in Figure 4. The gain of all four filters is equal to 1 at $\omega =\omega_{0}$, so $k_{pd}=1$. The error of the PD is now

(11) $$\epsilon =v_{\beta_{N}}\cdot v_{o_{\alpha }}-v_{\alpha_{N}}\cdot v_{o_{\beta }}\approx k_{pd}\cdot sin\left(\hat{\theta }-\theta_{i}\right)\approx \left(\hat{\theta }-\theta_{i}\right)$$

The closed-loop transfer function is now equal to

(12) $$H_{CL}\left(s\right)=\frac{\hat{\theta }\left(s\right)}{\theta_{i}\left(s\right)}=\frac{K_{p}s+K_{i}}{s^{2}+K_{p}s+K_{i}}$$

It could be presented using this canonical form:

(13) $$H_{c}\left(s\right)=\frac{2\xi \omega_{n}s+\omega_{n}^{2}}{s^{2}+2\xi \omega_{n}s+\omega_{n}^{2}},$$

with

(14) $$\omega_{n}=\sqrt{k_{i}}\;\;and\;\;\xi =\frac{k_{p}}{2\omega_{n}}$$

Therefore, the two parameters of the PLL, $k_{i}$ and $k_{p}$, could be calculated if ξ and $\omega_{n}$ are initialized using the following equations,

(15) $$k_{p}=2\xi \omega_{n}\;\;and\;\;k_{i}=\omega_{n}^{2}$$

In order to obtain more stability while estimating the frequency, the damping factor is set to ξ=2. Furthermore, to minimize the noise bandwidth, $\omega_{n}$ is set to $\omega_{c}/10$ with $\omega_{c}$ representing the central angular frequency.

### 3.3. Online Resampling

**Figure 6:**
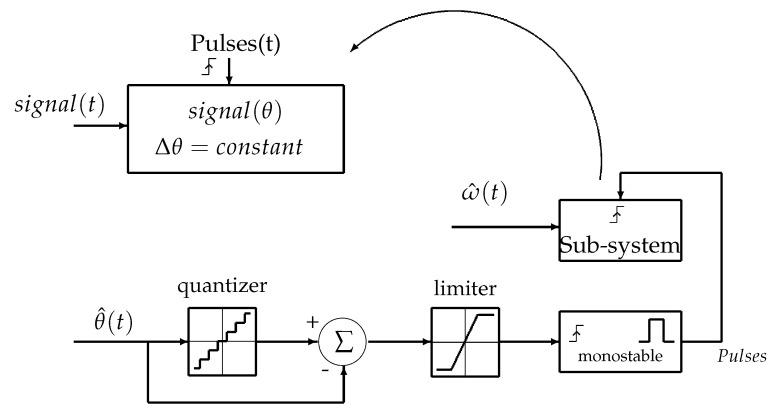
Block diagram of the online resampling.

Order Tracking (OT) consists of replacing the traditional temporal sampling of the signal containing fault informations with angular sampling. The signal resampling converts it from the time-domain (Δt) into the angle-domain, where samples are captured every rotor position increment Δθ. The Hardware Order Tracking (HOT) is a solution that provides electric impulses every angle increment using a sensor attached to the machine. The TOT provides those position impulses without adding any new sensors. It uses the estimated position vector $\hat{\theta }\left(t\right)$ and transforms it to an equally spaced vector with constant Δθ. To do that, many offline interpolation algorithms are used in [27,28,29,30,31,32]. In this paper, a new online (real-time) algorithm is used. The proposed algorithm is part of the TOT family as resampling does not need a speed sensor to be implemented. This algorithm is an alternative of a sensor directly supplying digital data sampled at an angle. The block diagram is shown in Figure 6. A quantizer is used to detect angle steps Δθ of the estimated phase $\hat{\theta }\left(t\right)$, then pulses are generated at every angle step using a monostable. The pulses vector will be used as a trigger to convert the time-dependent signal to a new position-dependent one which spectrum will remain stationary even with speed varying conditions.

The PLL described in Section 3.1 provides both the estimated speed $\hat{\omega }\left(t\right)$ and mechanical position $\hat{\theta }\left(t\right)$. The fault signature is looked for in $\hat{\omega }\left(t\right)$ and the position is used for resampling. In the following, the complete procedure is tested on the laboratory test bench.

## 4. Experimental Results

### 4.1. Test Bench Description

**Figure 7:**
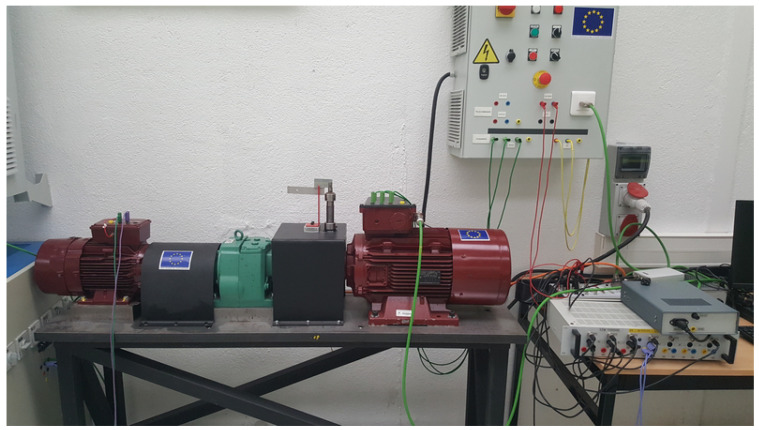
Experimental set-up.

A wind turbine test bench is used to verify the proposed method. As shown in Figure 7, two Permanent Magnet Synchronous Machines of 8 kW are used.

**Figure 8:**
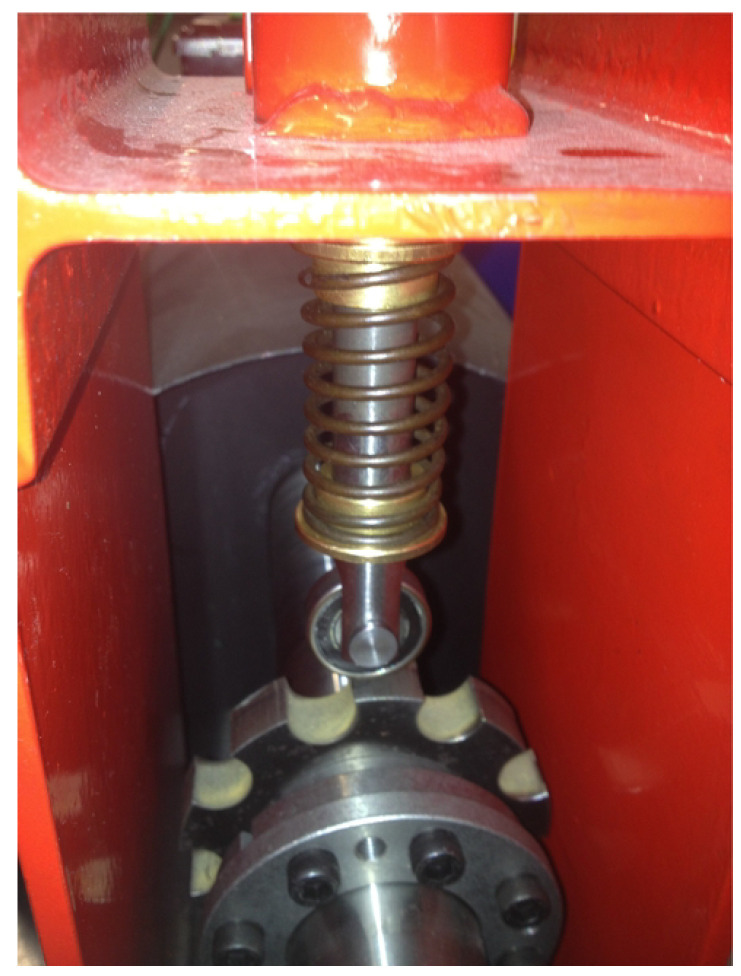
Fault emulator.

The machines have P=4 pole pairs. The PMSG is driven by a PMSM through a gearbox with 4.57 ratio. The motor is controlled through a variable speed drive. The generator is connected to a passive load. Current measurements are collected via a dSPACE-DS1104 acquisition card with a sampling frequency $f_{s}=10$ kHz. The rotor position is also collected to compare the results. In Figure 8, a mechanical system is designed to simulate the fault. As mentioned before, mechanical faults generate either variations of torque or variations of eccentricity. The emulator makes it possible to simulate the resistance torque variations (for low impacts) and the air gap variations (for strong impacts). The system is mounted between the motor and the gearbox (low speed part). A vertical roller impacts a sprocket of 9 teeth. This interaction generates 9 impacts per turn. The vertical axis could be controlled to change the force of the generated fault.

### 4.2. Results

**Figure 9:**
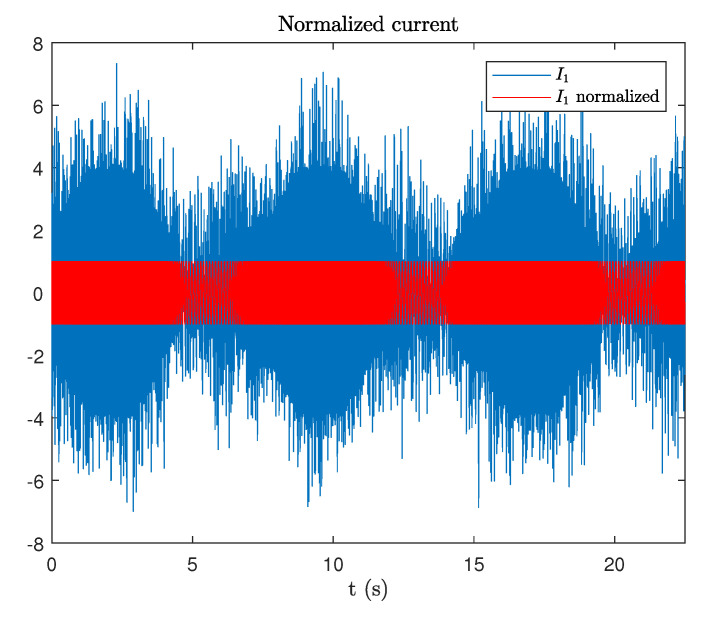
Normalization of the input signal $i_{1}$.

In order to verify the method, a test is realized with variable speed. The electrical frequency of the generator current changes approximately 30 Hz between 10 Hz and 50 Hz with a cycle of 7.5 s. In this test, the data of three cycles with total duration of 22.5 s are collected. Using the current $i_{1}$, an amplitude normalization is performed at the input stage, see Figure 9.

**Figure 10:**
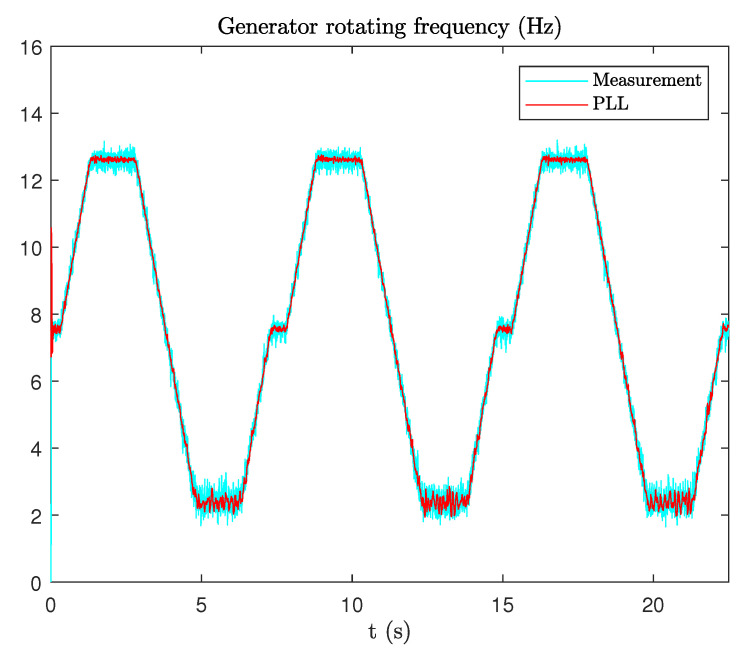
Estimation of rotating mechanical frequency.

After normalization, the PLL will estimate the phase $\hat{\theta }\left(t\right)$ and the electrical angular frequency $\hat{\omega }\left(t\right)$ using the normalized current. The central frequency of the PLL is set to $\omega_{c}=2\pi \cdot 30$ rad/s. $k_{p}$ and $k_{i}$ are set using Equation (15). For ξ=2 and $\omega_{n}=\omega_{c}/10$, the parameters are set to $k_{p}=75$ and $k_{i}=355$, respectively. As mentioned before, P=4, so the mechanical rotating frequency of the generator is 1/4 its electrical frequency, which means it will change between 2.5 Hz and 12.5 Hz. The estimated frequency and the measured one are shown in Figure 10.

**Figure 11:**
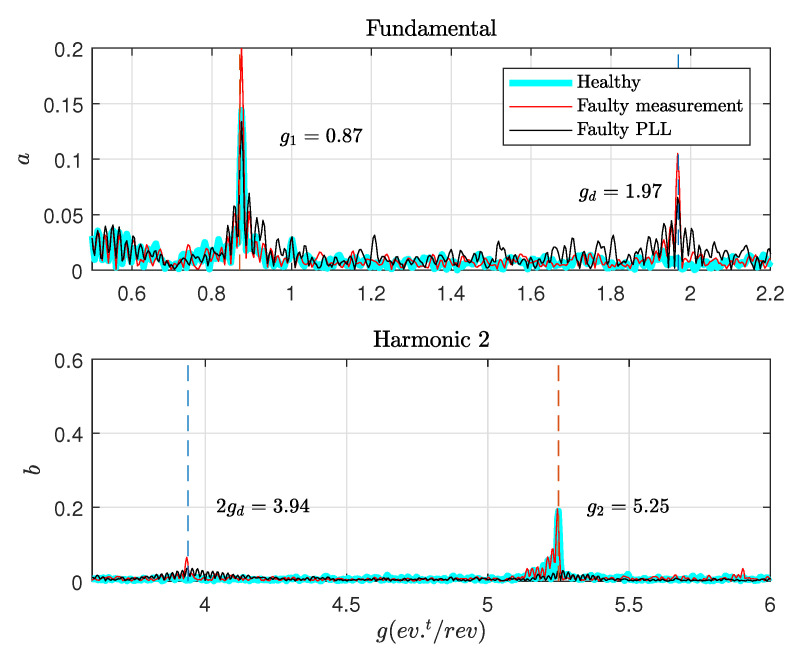
Angular spectrum of mechanical frequency.

The estimated phase $\hat{\theta (t)}$ is then used to resample the estimated frequency $\hat{\omega }\left(t\right)$. The online resampling is executed with an angular step $\Delta \theta_{s}=0.2$ rad; the Fast Fourier Transform FFT of the new signal $\hat{\omega }\left(\theta \right)$ is calculated. The angular spectrum for both healthy and faulty measurements are shown in Figure 11, it is represented in events per rotor revolution. The default generates nine impacts per revolution at low speed side. On the other side of the gearbox (generator side), the number of impacts generated is $g_{d}$ = 9/4.57 = 1.97 impacts/rev. The two components $g_{1}$ and $g_{2}$ are related to the gearbox, they are presented in healthy and faulty conditions.

**Figure 12:**
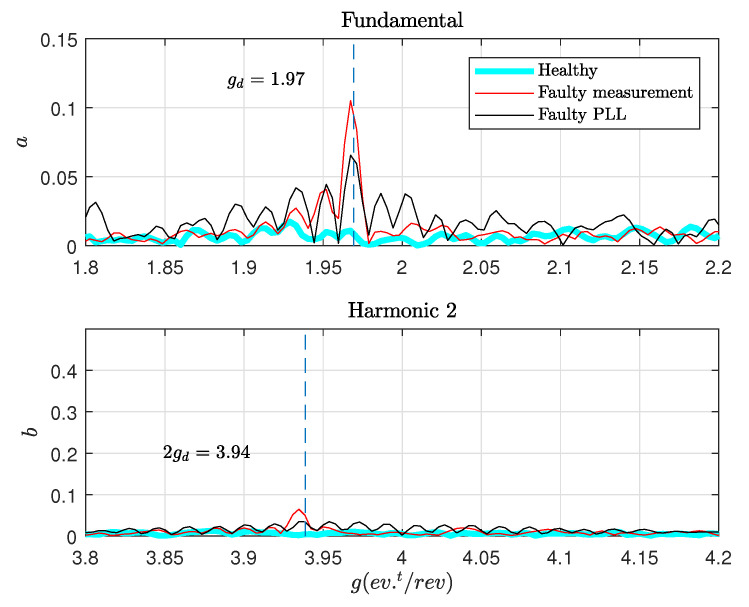
Zoom around fault components in angular spectrum.

In Figure 12, a zoom around the fundamental and the second harmonic $g_{d}$ and 2$g_{d}$ is shown. The fault signature is correctly detected at 1.97 event/revolution. The second harmonic at 3.94 event/rev could be detected but it is attenuated due to the filters used at the input of the PLL.

In this section, the modified PLL is tested on data with variable speed. The rotating speed is estimated as well as the rotor position. The estimated speed is then resampled in order to obtain an angle-dependent signal. A classical FFT shows that the angular spectrum is stationary and the components are no longer shifting with speed variations. It is shown that fault components are identified in angular spectrum. In the next session, a statistical-based method is used for fault detection.

## 5. Diagnosis Protocol: Statistical Approach

### 5.1. Protocol Principle

To create a diagnosis protocol, an alarm should be triggered automatically when a fault appears. Then, a threshold of a specific signature should be defined. To achieve that in a noisy environment, the statistical approach is a robust and reliable solution. In [33,34], a statistical diagnosis approach is proposed for speed-varying conditions. This approach proposes two phases: learning phase and diagnosis phase. The fault signature chosen is the amplitude of the fault corresponding frequency in the angular speed signal:

(16) $$S_{fault}=|{\hat{f}}_{m}\left(f_{d}\right)|$$

During the learning phase, a statistical reference of healthy state is created. To do that, $N_{ref}$ recordings are registered. Then, the fault signature $S_{fault}\left(k\right)$ of every recording is determined. The statistical features mean μ and standard deviation σ are calculated as follows,

(17) $${\hat{\mu }}_{ref}=\frac{1}{N_{ref}}\sum_{k=1}^{N_{ref}}S_{fault}\left(k\right)$$

(18) $${\hat{\sigma }}_{ref}=\sqrt{\frac{1}{N_{ref}-1}\sum_{k=1}^{N_{ref}}{\left(S_{fault}\left(k\right)-{\hat{\mu }}_{ref}\right)}^{2}}$$

In order to make the signature independent of machine type, a reduced centered signature is defined as follows,

(19) $$S_{fault,RC}\left(k\right)=\frac{S_{fault}\left(k\right)-{\hat{\mu }}_{ref}}{{\hat{\sigma }}_{ref}}$$

After creating the normalized reference, a threshold could be determined to lunch automatically a faulty state alarm. A probability of 1% is inspired from the Gaussian distribution using the following equations,

(20) $$P(S_{fault,RC}\left(k\right)>t_{1\%})=0,01$$

This probability is calculated as follows,

(21) $$P(S_{fault,RC}\left(k\right)>t)=1-\Phi \left(t\right)$$

The alarm threshold for a probability of 1% means that when the alarm is launched declaring a faulty state, there is 1% chance of it being a false alarm. This probability could be changed depending on the used application (0.1% for example). The diagnosis phase could now started after setting the alarm threshold.

This approach is simple when the rotation speed is constant. It becomes more complex for variable speed conditions. The proposed solution in [34] is to divide the torque-speed into *N* different zones. A normalized reference and alarm threshold are created for every zone during the training phase. After that, when the diagnosis phase starts, the torque and the speed of the machine are calculated to allow the determination of a specific functioning zone of the machine in the torque-speed plane. The decision is made according the this specific zone.

In this paper, it is shown that order tracking method creates a stationary spectrum (events per revolution). Therefore, the statistic features are usable even with variable speed conditions. Which allows us to use the simple statistical approach without the segmentation solution. In the following section, the experimental results of this approach are shown.

### 5.2. Results

**Figure 13:**
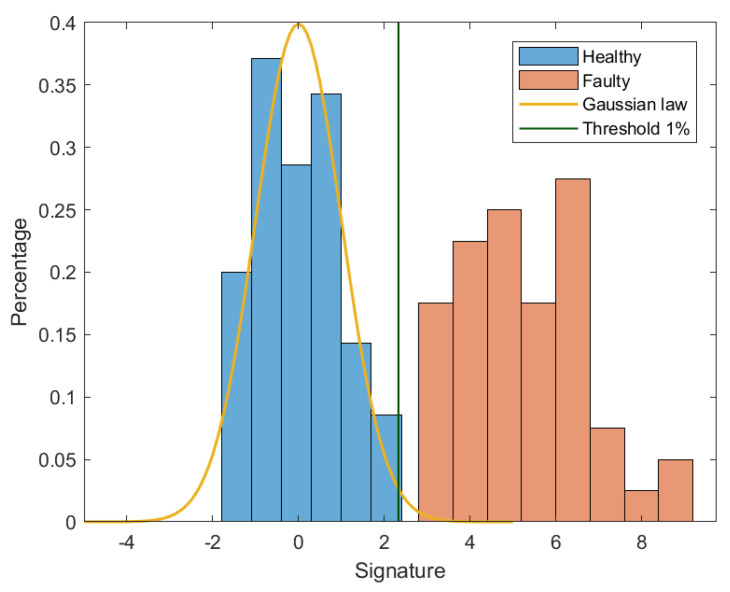
Histogram distribution of fault signatures using 1 cycle.

**Figure 14:**
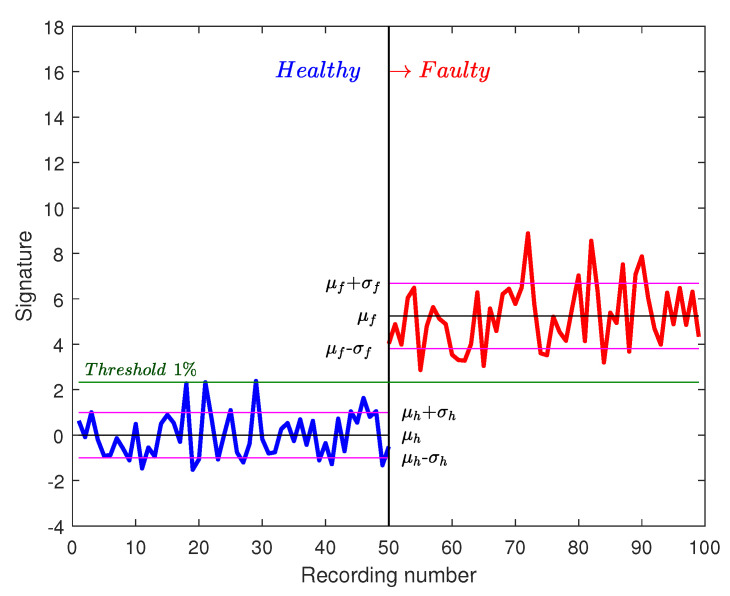
Fault signatures using 1 cycle with 50 recordings.

In order to validate the statistical approach, the same test bench of Section 4 with the same conditions are used. In the training phase, $N_{ref}=50$ recordings are collected. Each recording represents one speed cycle with a duration of 7.5 s. The fault signatures $S_{fault}$ are calculated. The normalized reference is then created. The threshold of a probability of 1% equals 2.33. In Figure 13 and Figure 14, the distribution of normalized signatures and the threshold are shown. In the diagnosis phase, 50 other recordings are collected for faulty conditions.

**Figure 15:**
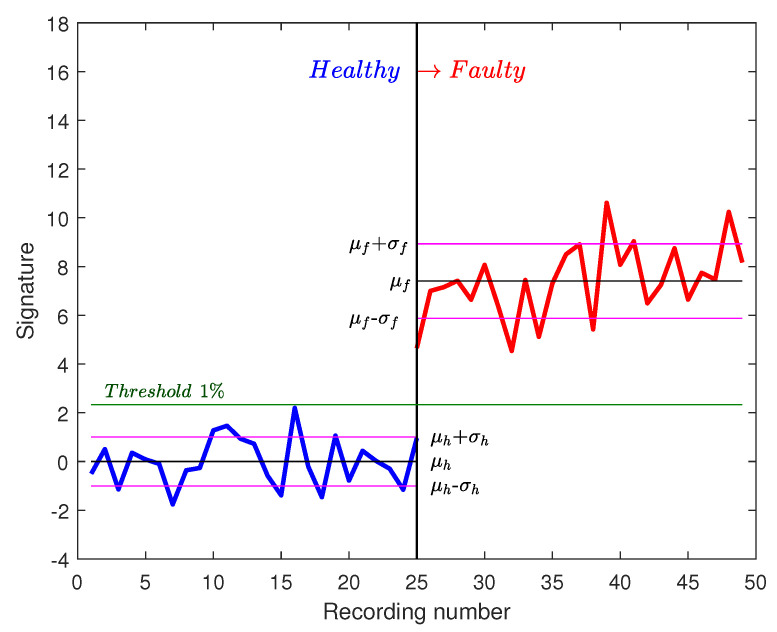
Fault signatures using 2 cycles with 25 recordings.

It is noted that the threshold separates healthy from faulty signatures. Those signatures are calculated with FFT on one speed cycle. In order to improve this approach and decrease the risk of launching false alarms, new signatures are calculated on two cycles for a duration of 15 s. In Figure 15, 25 healthy recordings of two cycles are shown as well as 25 faulty recordings. It is noted that the difference between healthy and faulty signatures is bigger. A more reliable threshold could be defined now (0.1% for example).

## 6. Conclusions

In this article, we have implemented a Tacholess Order Tracking technique from a single current measurement. The proposed method makes it possible to estimate both the instantaneous speed containing the fault signatures and the mechanical position used for resampling. The objective was to achieve a system providing a resampled signal in real-time. For this, an original procedure has been proposed. The tests were carried out on synchronous machines, which is a simple case. Indeed, the speed is directly proportional to the electrical frequency. Future work concerns the extension of this method to asynchronous machines where slip must be taken into account. Furthermore, the objective is to produce an innovative industrial product. Additional work will be necessary to study the transition of algorithms from our Matlab/Simulink platform to an embedded system.
